# Effects of a New Nano-Silver Fluoride-Containing Dentifrice on Demineralization of Enamel and *Streptococcus mutans* Adhesion and Acidogenicity

**DOI:** 10.1155/2018/1351925

**Published:** 2018-05-08

**Authors:** Joás Araújo Teixeira, Amitis Vieira Costa e Silva, Valdeci Elias dos Santos Júnior, Paulo Correia de Melo Júnior, Manuela Arnaud, Maria Goretti Lima, Miguel Angel Pelagio Flores, Thayza Christina Montenegro Stamford, José Ricardo Dias Pereira, Andréa Gadelha Ribeiro Targino, André Galembeck, Aronita Rosenblatt

**Affiliations:** ^1^Faculty of Dentistry, University of Pernambuco, No. 1650 Av. Gal. Newton Cavalcanti, 54756-020 Camaragibe, PE, Brazil; ^2^Center of Strategic Technologies of Northeast, Campus MCTI Nordeste, No. 01 Av. Prof. Luiz Freire, 50740-540 Recife, PE, Brazil; ^3^Federal University of Pernambuco, Cidade Universitária, No. 1235 Av. Prof. Morais Rego, CEP 50670-901 Recife, PE, Brazil; ^4^Department of Dentistry, Universidade Federal da Paraiba, 50670-901 João Pessoa, PB, Brazil

## Abstract

An experimental dentifrice containing nano-silver fluoride (NSF) and a sodium fluoride (NaF) toothpaste were tested *in vitro*, against *S. mutans*, to evaluate the minimal inhibitory concentration (MIC), minimal bactericidal concentration (MBC), antiadherence, antiacid, enamel microhardness, and OCT. The microdilution technique was used to determine the MIC and MBC. Fragments of deciduous enamel were treated with dentifrice slurries, containing bacterial suspension and PBS-treated saliva. The quantification of the microorganisms that adhered to the enamel was determined after 24 hours of incubation, and media pH readings were performed after 2 hours and 24 hours. Deciduous teeth were evaluated for microhardness and OCT during 14 days of pH cycling. Data were statistically analyzed using Student's *t*-test, Mann–Whitney *U* test, ANOVA, and Tukey tests at 5% of significance. Dentifrices containing NSF presented a lower MIC and higher statistically significant results compared to NaF dentifrices with respect to preventing bacterial adhesion and pH decreases. NSF and NaF dentifrices showed the same ability to avoid enamel demineralization corroborated by the OCT images. The NSF formulation had a better antibacterial effect compared to NaF dentifrices and similar action on the demineralization of enamel indicating their potential effectiveness to prevent caries.

## 1. Introduction

Mutans streptococci produce short-chain acids which dissolve the hard tissue of teeth. They are highly acidogenic and metabolize sucrose to synthesize insoluble extracellular polysaccharides which enhance their adherence to the tooth. For this reason, *Streptococcus mutans* is considered to be the most cariogenic bacteria [1].

*S. mutans* and *S. sobrinus* are the main microorganisms involved in the caries process. However, *S. mutans* is more cariogenic because it produces cell-surface proteins that help it to adhere to the dental surface [2]. In addition, *S. mutans* is able to metabolize sucrose, glucose, fructose, and maltose in cellular glucogen-like polysaccharides [3], as well as acids that lead to demineralization of the enamel surface that leads to the induction of caries lesions [2].

The evidence supporting the efficacy of fluoride in preventing caries in children and adolescents is indisputable, and since tooth brushing with fluoridated dentifrices is the easiest way to deliver fluoride into the mouth, it is widely used by all ages. The declining prevalence of caries in the last 30 years in Western countries has been attributed to the widespread use of fluoride products [4]. Fluoride inhibits the demineralization of enamel by changing the critical pH for dissolution of Ca^2+^ and PO_4_^3−^ in bacterial biofilm and adsorbing the apatite crystal surfaces, where it replaces Ca^2+^ to form acid-resistant mineral fluorapatite with low solubility [5]. Moreover, fluoride causes inhibition of bacterial activity [6] and acid production of *S. mutans* [7].

The antibacterial activity of silver on a wide range of microorganisms has been well known since ancient times and, at low concentrations, is not toxic to human cells [8]. Silver has a high chemical affinity for compounds containing nitrogen, sulfur, and phosphorus, so it has been suggested that the inhibitory power of silver ions is due to its interaction with the thiol groups of proteins and the phospholipid portion of the bacterial membrane [9]. When formed into nanoparticles, silver interacts more intensely with other organic and inorganic molecules due to its greater surface area and acts on the bacterial membrane to alter its permeability, thus causing its rupture. Inside the cell, silver interacts with nucleic acids to prevent the cell replication process [8–10].

*In vitro* studies have successfully tested the bactericidal action of silver nanoparticles (AgNPs) on *S. mutans*. Espinosa-Cristóbal et al. [11] verified the bactericidal action of spherical AgNPs of different sizes (8.4, 16.1, and 98 nm) on *S. mutans* (ATCC 25175). The results showed that bactericidal properties depended on the size of the particles: particles with smaller diameter exhibited lower inhibitory concentrations than particles with larger diameter. AgNPs at a concentration of 4.86 ± 2.71 *μ*g/mL are already sufficient to inhibit the growth of *S. mutans* [12] even when organized as biofilm, suggesting that they can be used for the prevention and treatment of caries [13].

Nano-silver fluoride (NSF) was developed in order to be a new anticarie agent. It is a colloid based on chitosan, silver nanoparticles, and fluoride which prevents and arrests dental decay in children [14]. This new silver agent presents no substantial risk for use in living organisms or harm to human health [15, 16], and its caries arresting property was described in a controlled clinical trial. Unlike silver diamine fluoride, NSF did not show distinctive tissue darkening of silver ion oxidation when in contact with the teeth [17].

The effectiveness of NSF in the arrestment of caries is attributed to the synergistic actions of both silver nanoparticles and fluoride. However, the mechanism of arrestment is not clear. Moreover, its preventive action on the sound enamel has not yet been reported. In this study, NSF was synthesized and formulated into a dentifrice, and its effects on reducing the production of acid and adhesion of *S. mutans* AU 159 and on reducing mineral loss during pH decline by microhardness analysis and optical coherence tomography (OCT) were evaluated *in vitro*.

## 2. Materials and Methods

This study was approved by Federal University of Paraíba Ethics Commitee (protocol number 0469/15 and CAAE 48033215.0.0000.5188) in accordance with the World Medical Association Declaration of Helsinki.

### 2.1. Bacteria

In the present study, a standard strain *S. mutans* AU159 was used. Initially, the inoculum was grown in brain heart infusion broth (BHI) with 2% sucrose at 37°C for 24 hours. Subsequently, standardization was performed spectrophotometrically until it reached 4 on the McFarland scale.

### 2.2. NSF

**Figure 1:**
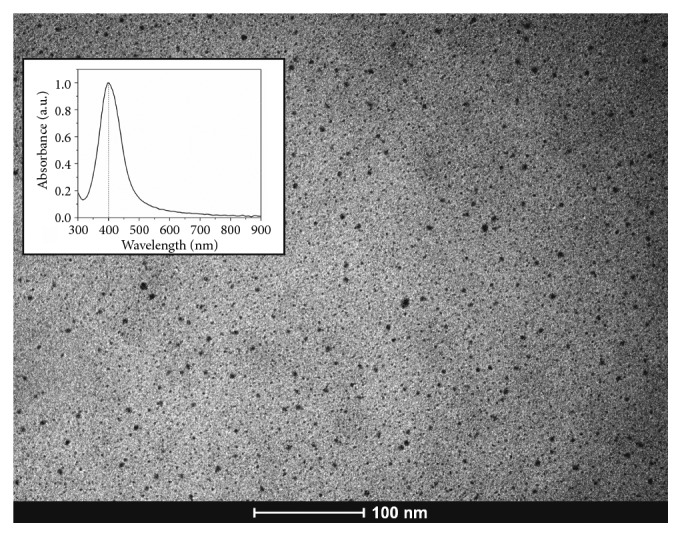
Silver nanoparticles in the NSF validated by the transmission electron microscopy image and UV-Vis spectrophotometry.

To prepare NSF, 1 g of chitosan was dissolved in 200 mL of 2% (V/V) acetic acid solution. The solution was stirred overnight and then vacuum filtered. Later, it was added to 60 mL of chitosan solution and placed in an ice bath while stirring. Then, 4 mL of 0.012 mol/L silver nitrate solution (AgNO_3_) was added and incubated for 30 minutes before adding sodium borohydride (NaBH_4_). A mass ratio of 1 : 6 between AgNO_3_ and NaBH_4_ was maintained by adding the solution dropwise. After 45 minutes in the ice bath, the colloid was removed from the bath and allowed to reach room temperature before being stored at 4°C. AgNPs were validated using UV-Vis spectroscopy that showed a peak at 400 nm wavelength while transmission electron microscopy (TEM) showed spherical and monodisperse particles that were 8.7 ± 3.1 nm in diameter (Figure 1).

### 2.3. Preparation of Dentifrices

A dentifrice formula containing NSF (test dentifrice) was prepared and is summarized in Table 1. To evaluate the antibacterial effects of NSF, control dentifrices without NSF were also made. All dentifrices were made by the CETENE (Centro de Tecnologias Estratégicas do Nordeste) laboratory (Brazil). The dentifrice slurry was prepared by mixing dentifrice and sterilized deionized water at a 1 : 3 (w/w) ratio. After centrifugation, the supernatant was passed through a 0.2 *µ*M membrane pore diameter filter and used as the primary solution.

Determination of Minimum Inhibitory Concentration (MIC) and Minimum Bactericidal Concentration (MBC)

Flat bottom 96-well microplates were used for the microdilution experiments. The microplates were prepared so that each well had a final volume of 100 *μ*L with varying volumes of culture medium (BHI). The dentifrices were maintained at a constant 20 *μ*L of bacterial inoculum. The assay was carried out in duplicate.

The microplates were incubated at 37°C for 24 h before 30 *μ*L of resazurin was added to each well. The microplate was incubated at 37°C for 1 h before the color change analysis was performed.

Resazurin is a blue oxide-reducing indicator compound which in the presence of viable cells is oxidized to resorufin, a red substance. Thus, the blue stain indicates the absence of microbial growth, while pink and purple stains indicate the presence of viable cells.

After determination of the MIC, the sample with the lowest concentration which contained visible growth and all samples in which there was no visible growth were cultured in Petri dishes containing BHI agar. 10 *μ*l of the microplate wells (without resazurin) were transferred to the Petri dishes and were incubated at 37°C in microaerophilia for 24 h. The MBC was determined by the lowest concentration of the test substance at which there was no microbial growth.

### 2.4. Adherence Test

The bacterial adherence and acid production assays were carried out using a modified version of the Kim et al. method [18]. Twenty autoclaved enamel 5 × 5 mm fragments were divided into 2 groups with 10 units: the test group (NSF dentifrice) and the control group (NSF-free dentifrice). The enamel samples were obtained from caries-free deciduous molars extracted for natural exfoliation.

A bacterial suspension and PBS-treated saliva solution were prepared wherein 200 *µ*L of the bacterial inoculum was transferred to 20 test tubes containing 1 mL of sterile PBS with 10% sucrose and 1 mL of saliva. Saliva used in the experiment was collected from volunteers aged 20 to 30 years old who were not taking any systemic medications, in accordance with the Stamford et al. method [19]. Saliva was centrifuged (1,200 ×g) for 15 minutes and then heated at 60°C. The supernatant (treated saliva) was stored at 4°C for later use.

The 20 enamel fragments were placed into a 24-well microplate and immersed in 2 mL of dentifrice slurry, according to their group, for 1 minute. The enamel fragments were removed from the wells, washed with sterile deionized water, and then placed into the test tubes containing the bacterial suspension and PBS-treated saliva. The fragments were kept in this fluid for 2 h at 37°C. The fragments were then transferred to Eppendorf tubes containing 1 mL of sterile saline (0.9% sodium chloride) and then sonicated for 30 sec at 50 W to release the bacteria adhered to the enamel. Saline solution containing the bacterial suspension from the dental enamel was sequentially diluted (10^−1^ to 10^−6^) and cultured in Petri plates with mitis salivarius agar broth at 37°C for 24 h to allow for the counting of colony-forming units (CFU/mL). This step was performed in triplicate. The quantification of the microorganisms that adhered to dental enamel was determined by direct reading of CFU and the subsequent calculations of the adsorption inhibition percentage for each test solution.

### 2.5. Acidogenicity

The 20 enamel fragments were placed into a 24-well microplate and immersed in 2 mL of dentifrice slurry, according to their group, for 1 minute. The fragments were removed from the wells, washed with sterile deionized water, and placed into test tubes containing the bacterial suspension and PBS-treated saliva. The fragments remained in the tubes for 24 h at 37°C. The pH was directly determined in bacterial growth media using a pH meter. The pH readings were performed in triplicates at 2 hours and again at 24 hours.

### 2.6. Evaluation of NSF Dentifrice Performance in Enamel Remineralization

The samples comprised 48 deciduous teeth free of caries. Natural exfoliated molars were used for preparation of the specimens for microhardness and OCT tests. The teeth were cleaned, autoclaved, and stored in distilled water under refrigeration. The roots were sectioned 1-2 mm below the cementoenamel junction and the crowns were embedded in acrylic resin using a PVC mold leaving 5 × 5 mm surface exposed. After polymerization, each enamel surface was flattened by polishing with waterproof sandpapers (120, 400, and 600 grit) under water-cooling for 20 seconds. Finally, the surfaces were polished with diamond paste at low speed. The specimens were randomly divided into three groups (Table 2) of 16 units. The groups were composed as follows: a test group (NSF dentifrice), a positive control group (NaF dentifrice), and a negative control group (deionized water). The specimens were kept in phosphate-buffered saline solution (8 g/L NaCl; 2 g/L KCl; 2 g/L Na_2_HPO_4_; 2 g/L KH_2_PO_4_; pH = 7.0) for enamel ionic restoration until further microhardness tests were performed.

Microhardness tests were performed using a Shimadzu tester and a Vickers indenter (Shimatzu Corporation, Kyoto, Japan) at a load of 50 g for 10 seconds. The initial microhardness for each specimen was determined as the baseline. After the pH cycling, each sample was again analyzed to determine its final microhardness. Three indentations were made on each sample spaced 100 *µ*m apart.

The pH cycling tests were performed as described by Stookey et al. [20]. Demineralization (1) and remineralization (2) solutions were used:

1. 2.0 mmol/L Ca; 2.0 mmol/L P; 75 mmol/L acetate buffer (pH = 4.4)
2. 1.5 mmol/L Ca; 0.9 mmol/L P; 130 mmol/L KCl; 20 mmol/L sodium cacodylate buffer (pH = 7.0)

The samples were divided and treated for 1 minute with one of the following:

- 100 *µ*L of the NSF dentrifice slurry
- 100 *µ*L of the NaF dentifrice slurry (positive control)
- 100 *µ*L of deionized water (negative control)

Each sample was rinsed with deionized water and dried with absorbent paper. This treatment was performed twice a day immediately before each sample was left to soak in the demineralization and remineralization solutions. In individual vials, each sample was left to soak in 40 mL of demineralization solution for 6 hours at 37°C. After this period, the samples were rinsed with deionized water and left to soak in 20 mL of remineralization solution for 16 hours at 37°C. This cycle was repeated for another 13 days, providing 14 days of total cycle time. The samples were stored in remineralization solution for approximately 48 hours.

A commercial OCT system model (Callisto–Spectral Domain OCT System, Thorlabs Inc., New Jersey, USA) was used to analyze the samples before and after the pH cycling process, as detailed by Mota et al. [21]. The Callisto uses a superluminescent diode laser operating at a 930 nm central wavelength as a light source, with a 100 nm spectral bandwidth and a 3 mW maximum optical power. This model images samples at a 7 *μ*m axial resolution when immersed in air and a 5.3 *μ*m axial resolution when immersed in water. The transverse resolution does not depend on the background, being set at 8 *μ*m. The axial scan rate is 1.2 kHz, which captures two frames per second with a 105 dB sensitivity.

The Callisto SD-OCT captures data in a matrix of 512 lines × 2,000 columns. The A-scan mode projects *Y*-axis data as dependent on the deep penetration of light, limited to 1.7 mm. The B-scan mode creates proper 2D-OCT images, which are composed of all 2,000 A-scans captured along a width up to 6 mm, corresponding to 1.7 mm maximum depth penetration (in air). A complimentary 3D mode was composed of B-scans captured in a sequence of 250 *μ*m steps, until the complete mapping of the surfaces and subsurfaces of the samples was achieved. Tridimensional images allow the user to visualize B-scans along the *XY*, *XZ*, and *YZ* planes.

### 2.7. Statistics and OCT Images Analysis

All data were converted to percentages and were then analyzed by SPSS 13.0.

The statistical analysis included the Kolmogorov–Smirnov test for evaluating the normality and homogeneity of the data.

The inhibition percentage of bacterial adsorption for each sample was calculated: 100 ((number of adsorbed cells to enamel in the control group − number of adsorbed cells in the test group)/number of adsorbed cells in the control group). The initial and final pH values obtained for each sample were converted to percentages of pH variation (%pH = (100 (pH_*f*_ − pH_*i*_)/pH_*i*_)). The data were analyzed using Student's *t-*test for adsorption inhibition percentage and the Mann–Whitney *U* test for percentage of pH variation using a 5% threshold for statistical significance.

A microhardness test was conducted before and after pH cycling treatment of the enamel samples with dentifrices. The initial and final values of the microhardness obtained for each sample were first converted into the percentage of microhardness variation (%microhardness variation = (100 (microhardness_*f*_ − microhardness_*i*_)/microhardness_*i*_)). The percentage of microhardness variation was analyzed by the analysis of variance (ANOVA) and Tukey tests for a 5% significance level.

The OCT results were processed with ImageJ software (National Institute of Health, https://imagej.nih.gov/ij/) [22] with a computational routine that averaged 50 user-selected A-scans. In fact, the choice of the region of interest (ROI) is important because it ensures that the analysis is meaningful. For this, we chose the central region of the OCT images at the specimens' flat surface. This allowed the depth of OCT signal penetration to be similar to the 50 A-scans. The demineralization and remineralization processes affect the profile of the OCT signal, at a certain depth, in the sample. This method allows qualitative analysis by examining the changes that occur in the optical properties of the enamel by comparing the integrity of the enamel surface and the measurement of the volume loss for the tissue, after pH cycling, with the initial image.

## 3. Results

### 3.1. MIC and MBC

The well containing 60 *μ*L of the NSF dentifrice slurry, 20 *μ*L of culture media, and 20 *μ*L of bacterial inoculum did not show bacterial metabolism. To determine the final concentration, which is the MIC, the expression *C*_1_ *V*_1_ = *C*_2_ *V*_2_ was used for calculations, where *V*_2_ is 100 *μ*L. Because the slurry was the result of the 1 : 3 dilution of the dentifrice in water, the NSF concentration decreased from 200 ppm to 50 ppm. Therefore, the MIC of the dentifrice test for *S. mutans* AU159 was 30 ppm.

The well containing NaF dentifrice that did not show bacterial metabolism also contained 60 *μ*L of the dentifrice slurry. The concentration of NaF in the dentifrice, which initially was 1,200 ppm, decreased to 300 ppm after its 1 : 3 dilution. Therefore, the MIC of the control dentifrice was 180 ppm for the same bacterium.

In relation to the MBC, based on the culture of the contents of the wells in the Petri dishes, neither the test nor the control dentifrices showed bactericidal action on *S. mutans* AU159.

### 3.2. Adherence Test

Data in Table 3 show the percentages of inhibition of the test and control dentifrices based on the adherence of *S. mutans* AU 159. The NSF-containing dentifrices outperformed the NaF-containing dentifrices in preventing bacterial adhesion to the dental surface with statistically significant difference (*p*=0.017).

### 3.3. Acidogenicity

Dentifrices containing NSF were able to prevent the pH decrease caused by the production of acids that results from *S. mutans* AU159 metabolism of sucrose in the media. All samples treated with the test dentifrices showed a mean value for the initial and final pH of 6.8, unlike samples treated with the control dentifrices which showed pH decreases after 24 hours of the bacterial incubation.  Table 4 displays the percentages of pH variation for the dentifrices, which showed statistically significant differences (*p* < 0.01).

### 3.4. Enamel Surface Microhardness

The variation in the percentage of hardness values presented significant differences between samples (averages followed by different letters in Table 5 are significantly different from each other *p* < 0.001). Accordingly, there was no statistically significant difference between the NSF dentifrice and the NaF dentifrice, and both showed the same ability to avoid demineralization in the samples. However, there were significant differences between the negative control solution compared to the NSF and NaF dentifrices.

### 3.5. OCT Images

**Figure 2:**
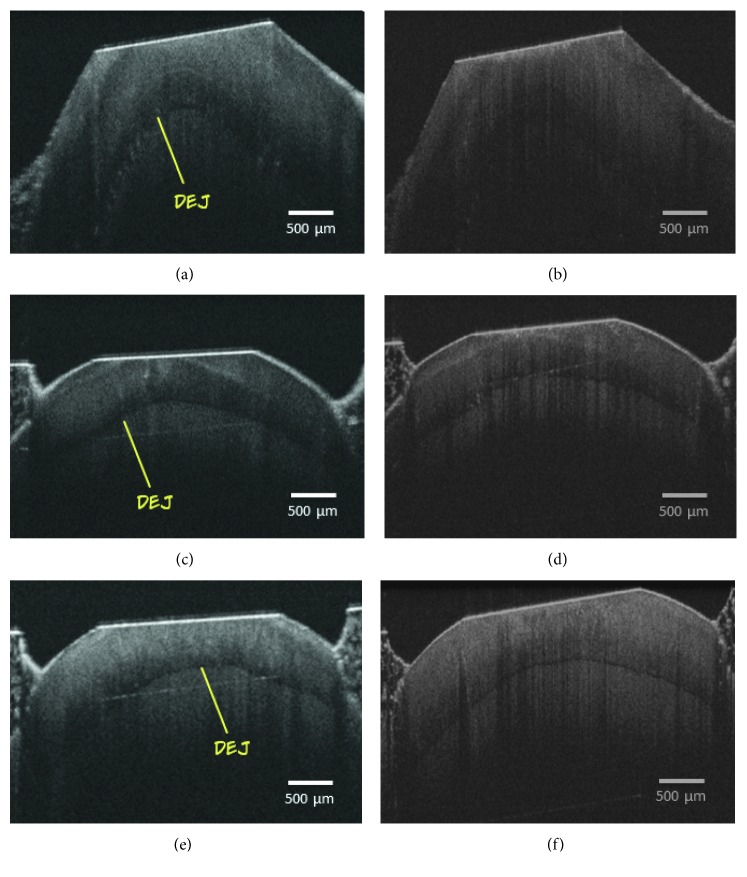
OCT images (a), (c), and (e)–negative control, NaF dentifrice, and NSF dentifrice groups, respectively, at *T*_*i*_. Images (b), (d), and (f)–negative control, NaF dentifrice, and NSF dentifrice groups, respectively, at *T*_*f*_. Arrows indicate the dentine enamel junction (DEJ).

**Figure 3:**
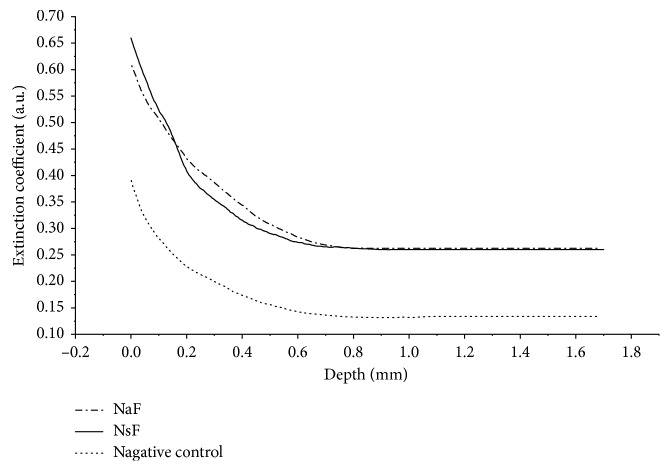
Exponential decay of A-scans obtained from each dentifrice group–NaF, NSF, and the negative control group–at *T*_*f*_.

The OCT analysis was performed twice: at *T*_*i*_ and at *T*_*f*_. This is detailed in Materials and Methods.  Figure 2 shows the OCT images acquired from representative sample of the negative control (Figures 2(a) and 2(b)), NaF dentifrice (Figures 2(c) and 2(d)), and NSF (Figures 2(e) and 2(f)) at *T*_*i*_ and *T*_*f*_ phases. Nevertheless, by analyzing the exponential decay of the A-scans from each group (Figure 3), it is possible to identify differences in light propagation into the samples, especially at *T*_*f*_. Notice that the decay curves obtained from NaF and NSF show similar behaviors, while the negative control group has a lower extinction coefficient.

Already described in the literature [23–29], differences can be observed in the teeth submitted to a cariogenic challenge, as shown in Figures 2(a), 2(c) and 2(e) at *T*_*i*_ (healthy enamel) and Figures 2(b), 2(d) and 2(f) at *T*_*f*_ (after cariogenic challenge). For instance, in Figure 2(b) (*T*_*f*_ images), it is not possible to visualize the dentine enamel junction (DEJ). Probably, this is due to the higher backscattering of the demineralized enamel, which does not allow the light to reach the dentin.

## 4. Discussion

The novelty of this study is to enhance dentifrices with NSF in order to reduce the bacterial growth. Since tooth brushing is a simple and available method for home dental care, the addition of NSF in dentifrices may be an alternative approach to prevent caries. This study demonstrated that a novel dentifrice containing NSF might significantly improve antibacterial efficacy compared to conventional 1,200 ppm sodium fluoride-containing dentifrices.

Studies have been reported on the antibacterial action of AgNPs on *S. mutans* [11, 12, 15]. Fluoride which is a component of NSF acts as an antimicrobial agent against *S. mutans* [7] and also as the chitosan which was used as a carrier [30] to stabilize AgNPs [31, 32]. The MIC of the dentifrice containing NSF, 1/6th of that of the NaF dentifrice, suggests a synergistic antibacterial action between the AgNPs, chitosan, and fluoride components in the NSF preparation. Further study should be done regarding this potential synergistic effect. Although the dentifrice with NSF demonstrated bacteriostatic but not bactericidal actions, this is a positive factor in an ecological point of view, given that successful antimicrobial agents would be able to avoid the disruption of the natural and beneficial resident oral bacteria, which are essential for oral health [3]. For these reasons, the current literature reports on numerous attempts to control microorganisms by blocking their specific adherence or coaggregation mechanisms [33, 34].

Bacterial adhesion plays an important role in biofilm formation and the pathogenesis of caries since bacterial glucosyltransferase enzymes produce glucans which promote adhesion between cell-cell and cell-surfaces [35]. Silver ions have shown the ability to prevent bacterial biofilm formation by disrupting this adhesion [36]. With respect to *S. mutans*, the antiadherence properties of AgNPs are associated with the smaller particle size [37]. In this study, the silver particle size in NSF was smaller than those particles used by Espinosa-Cristobal et al. [11], which could be related to the significantly superior performance of the test dentifrices. Fluoride at concentrations up to 300 ppm is effective for controlling cariogenic biofilms and causes significant reductions in bacterial extracellular polysaccharides formation [38]. The dentifrices containing NaF demonstrated almost half of the beneficial action of the dentifrices containing NSF in preventing bacterial adhesion.

The acid production of the bacterium also serves as a virulence factor because the acid products formed during the metabolism of dietary carbohydrates are essential for the development of caries. Fluorides are effective for acidogenicity reduction on cariogenic biofilms [38] even at low concentrations of 10 ppm of NaF [7]. However, in this study, the dentifrices containing NaF were not able to prevent bacterial acid production. The percentages of pH variation in the culture media of control dentifrices varied negatively on average, approximately 28.53%, compared to the initial pH of the media which was 6.8 for all samples tested. In other words, all samples treated with the NaF-containing dentifrice showed a decrease in pH of the media at the end of the experiment. On the contrary, the NSF-containing dentifrice-treated samples started with pH 6.8 in the media and did not alter its pH until the end of the experiment. The antimicrobial NSF mechanisms of action may also explain those findings due to its penetration into the bacterial cell and metabolic impairment [8–10].

Although our results revealed the inhibitory effects of the NSF-containing dentifrice treatment on *S. mutans*, it is necessary to investigate if the treatment can affect the ability of *S. mutans* to organize the biofilm and to interfere in other dental caries–related bacteria in biofilms (multispecies biofilm models), which is a limitation of the present study.

Clinical development of dental caries involves the simultaneous demineralization and remineralization, and the effect of fluorides in those processes is well known. Zhi et al. [37] reported that silver ions (non-nanoparticles) may increase mineral density during the remineralization of enamel caries. However, silver ions have little effect on the prevention of enamel demineralization, unlike fluorides, which are deposited on the enamel surface as CaF_2_, which is inclined for remineralization [38]. This study focused on investigating silver nanoparticles and fluoride-containing dentifrice effect on preventing the formation of enamel demineralization damage, which also differs from the studies mentioned above because the enamel surfaces were submitted to a pH cycling challenge. Hence, the results showed a similar effect (nonstatistically significant difference) of both NSF and NaF dentifrices on enamel demineralization even though the NaF presented a lower percentage of microhardness variation. All enamel surfaces were submitted to a pH challenge for 14 days [20] and all samples showed some degree of demineralization; however, NSF and NaF were more effective on avoiding demineralization than the negative control samples. Furthermore, NSF dentifrice-treated samples did not show black stains like SDF dentifrice-treated samples [17]. Since silver particles were reduced to nanometer size, their chemical reactivity was altered, and AgNPs colloid was not able to stain the teeth black. These results are a satisfactory outcome because NaF is considered the reference standard for the prevention of enamel demineralization.

The OCT presents limitations in the study of great depths (dentin). In the presence of caries, greater scattering can further reduce image quality at greater depths [39]. However, the thickness of the enamel was about 1-2 mm, and it was quite appropriate for this study. Thus, based on the characteristics of the examined object, parameters such as central wavelength, bandwidth, and optical instrumentation must be adjusted to enable the advantages of this system overcome their disadvantages [22].

The OCT images presented in Figure 2 showed the light propagation from the enamel surface to the dentin at *T*_*i*_ and *T*_*f*_ phases in the NSF dentifrice, NaF dentifrice, and negative control groups. Figures 2(a), 2(c), and 2(e) shows all length of enamel layer, dentin enamel junction (DEJ), and dentin (*T*_*i*_ phase). In this sense, in *T*_*f*_ phase, the same can be seen in Figures 2(d) and 2(e). However, in Figure 2(b) of the negative control group, this distinction between layers is not visualized due to the enamel demineralization. In this situation, there is an intense increase in backscattering at this region, so that the light does not reach the deeper layers, the dentine area. This fact corroborates the current literature reports, strengthening the argument that in the presence of demineralization, there is a greater scattering due to the modification of the properties in demineralized enamel, which can further reduce the image quality at greater depths [22].

The present findings, presented in OCT images, corroborate the data found in the microhardness test of this study, regarding the ability of NSF to prevent demineralization of primary tooth enamel. This is an important outcome because deciduous enamel is more porous and permeable than the enamel of the permanent tooth, which implies a greater susceptibility to demineralization by the acid action of cariogenic bacteria. This increased permeability of the deciduous enamel favors the action of fluorides, which diffuses about 150 times more than permanent teeth [40, 41].

Based on these results, NSF dentifrice could be a potential agent to be used for self-care caries control and in public health programs. Silver nanoparticles do not stain teeth black, which is a great advantage over SDF, and NSF dentifrice showed better action than NaF dentifrice on the virulence of *S. mutans*.

## 5. Conclusion

The new dentifrice containing NSF had bacteriostatic action against dental microbia. It showed significant antiadherence and antiacidogenicity effects on *S. mutans* AU159 compared to dentifrices containing fluoride alone, and it prevented mineral loss on the enamel surface of the specimens during pH cycling, similar to NaF.

## Figures and Tables

**Table 1:** Dentifrice formulations with approximate percentages of ingredients.

Ingredient	NSF dentifrice	NaF dentifrice
Silica	20–30%	20–30%
Sodium dodecyl sulfate	1-2%	1-2%
Glycerin	20–30%	20–30%
Carbopol + hydroxyethyl cellulose	1-2%	1-2%
Flavorizing	0.5–1.0%	0.5–1.0%
Sodium saccharin	0.05–0.2%	0.05–0.2%
Sodium fluoride	1,200 ppm	1,200 ppm
Sodium pyrophosphate	0.25–1.0%	0.25–1.0%
AgNPs	200 ppm	0%
Chitosan	0.3–1.0%	0%
Deionized water	15–25%	15–25%

**Table 2:** Groups description for the microhardness test.

Groups	Compounds
Test	Silver nanoparticle (NSF) dentifrice
Positive control	Sodium fluoride (NaF) dentifrice
Negative control	Deionized water

**Table 3:** Percentage (mean ± standard deviation) of adsorption inhibition of *S. mutans* AU159 to enamel surface treated with the test and control dentifrices.

Dentifrice	Bacterial adsorption inhibition (%)
NSF	88.94 ± 31.32
NaF	41.06 ± 50.76

Kolmogorov–Smirnov test: *p*=0.124.

**Table 4:** Percentage of pH variation (mean ± standard deviation) of samples treated with test and control dentifrices.

Dentifrice	pH variation (%)
NSF	0 ± 0
NaF	−28.53 ± 1.24

Kolmogorov–Smirnov test: *p* < 0.01.

**Table 5:** Percentage (mean ± standard deviation) of Vickers microhardness variation of enamel treated with test and control dentifrices and negative control groups.

Groups	Enamel microhardness variation (%)
NSF dentifrice	−11.07 ± 19.94^(a)^
NaF dentifrice	−0.34 ± 26.16^(a)^
Deionized water	−77.95 ± 26.30^(b)^

Kolmogorov–Smirnov test: *p*=0.297. Different letters in superscript means statistically significant difference among variables.

## Supplementary Materials

Supplementary Materials Raw data of the microhardness test.
